# Reporter genes and transgenic *Trypanosoma cruzi* (Kinetoplastida, Trypanosomatidae): applications for screening new drugs against Chagas disease

**DOI:** 10.3389/fmed.2025.1591148

**Published:** 2025-05-20

**Authors:** Daniele Santana de Sousa Oliveira, Elis Dionisio da Silva, Fábio Farias Galvão Júnior, Danielle Maria Nascimento Moura, Policarpo Ademar Sales-Junior, Maria Carolina Accioly Brelaz-de-Castro, Valéria Rêgo Alves Pereira

**Affiliations:** ^1^Postgraduation Program in Therapeutic Innovation, Federal University of Pernambuco, Recife, Pernambuco, Brazil; ^2^Health and Biotechnology Institute, Federal University of Amazonas, Coari, Amazonas, Brazil; ^3^Immunopathology and Molecular Biology Laboratory, Aggeu Magalhães Institute (FIOCRUZ-PE), Department of Immunology, Recife, Pernambuco, Brazil; ^4^Parasitology Laboratory, Federal University of Pernambuco, Vitória de Santo Antão, Pernambuco, Brazil

**Keywords:** Chagas disease, *Trypanosoma cruzi*, transgenic parasite, reporter gene, drug screening

## Abstract

Chagas disease is considered a public health issue, especially in Latin America. To this date, the course of the infection caused by the parasite *Trypanosoma cruzi* is yet to be completely understood and the conventional treatment do not promote a cure in the chronic phase, meaning there is an urgent need to discover new drugs. The expression of reporter genes by transgenic parasites has become an important tool in the screening of new compounds, whether in the study of the parasite, in the development of *in vitro* and *in vivo* assays, or in the application of High-Throughput Screening utilizing compounds collections. This review sought to gather information about transgenic *T. cruzi* applications in screening studies of compounds with action specifically against Chagas disease, the reporter genes in use, besides the highlighted characteristics of each one by the literature, including the performed assays, evolutionary forms and techniques applied, aiming to facilitate the identification of the reporter gene system or research model whose characteristics best adapt to the needs of new studies, contributing to the decisions about a framework adaptable to the reality of laboratories, in the screening of potentially trypanocidal compounds.

## 1 Introduction

Chagas disease (CD) caused by the parasite *Trypanosoma cruzi* (Chagas, 1909) (1) (Kinetoplastida, Trypanosomatidae), is a neglected disease affecting over 7 million people globally and frequently results in chronic heart conditions (2). The disease in the host progresses through two main phases: an initial acute phase characterized by high parasitemia and often few or no symptoms, followed by a chronic phase where parasitemia becomes intermittent. This chronic phase can remain asymptomatic for years or eventually lead to severe neurological and cardiodigestive complications (3, 4). Currently available treatments rely on nitroheterocyclic drugs which unfortunately present significant toxicity (5).

Preventive control of CD involves reducing human-vector contact (6), implementing blood bank screening programs (7), ensuring strict food and beverage hygiene coupled with public awareness campaigns (8), and ongoing efforts to develop vaccines to prevent/slow disease progression (6).

Post-infection control relies on pharmacological treatment, limited to two drugs: nifurtimox (commercialized as Lampit™ by Bayer HealthCare AG, Leverkusen, Germany) and benznidazole (commercialized as Benznidazol LAFEPE® in Brazil by LAFEPE, and as Abarax® in Argentina by Maprimed/ELEA) (9).

These drugs are primarily effective during the acute phase of the disease, but their multiple adverse reactions are frequently reported, leading to treatment discontinuation in approximately 20% of patients, which reinforces the urgent need for new therapeutic options (9).

Ideally, a new drug should demonstrate efficacy for all phases of CD, improved safety and tolerability compared to benznidazole, no contraindications during pregnancy or age groups, and shorter treatment regimen (10).

Achieving this goal requires overcoming significant gaps in knowledge of parasite biology, its interactions with the host immune system (11), and current lack of tools to assess treatment efficacy (12).

Methodological challenges to develop anti-*T. cruzi* drugs are the absence of standardized protocols for *in vitro* and *in vivo* screening and the difficulty in extrapolating animal model findings to human disease (7); also insufficient comprehension of CD pathogenesis, chronicity, and tissue tropism (13).

Romanha et al. (14) established some criteria to new compound’s identification in preclinical studies, providing decision-making steps for progression to later testing stages, facilitating data comparison across research groups (9).

Transgenic parasites expressing reporter genes were emphasized in that document (14), and since then, the importance of genetically modified parasites in drug screening has grown (15), facilitating studies on cellular signaling and gene expression (16) while enabling rapid data quantification and reducing manual labor (17).

In murine models, screening of anti-*T. cruzi* compounds has become faster and more efficient through the use of recombinant fluorescent or luminescent parasites (18), complemented by imaging systems (19).

This review aims to identify transgenic *T. cruzi* used in drug screening for CD, *in vitro* and *in vivo*, employing techniques such as imaging and High-Throughput Screening (HTS). Strains were analyzed in relation to their life stage, reporter genes, advantages and limitations of each approach, in order to provide information that may guide toward efficient, rapid, and quantitative tools for the screening of potentially trypanocidal compounds.

### 1.1 The reporter system

**Figure 1:**
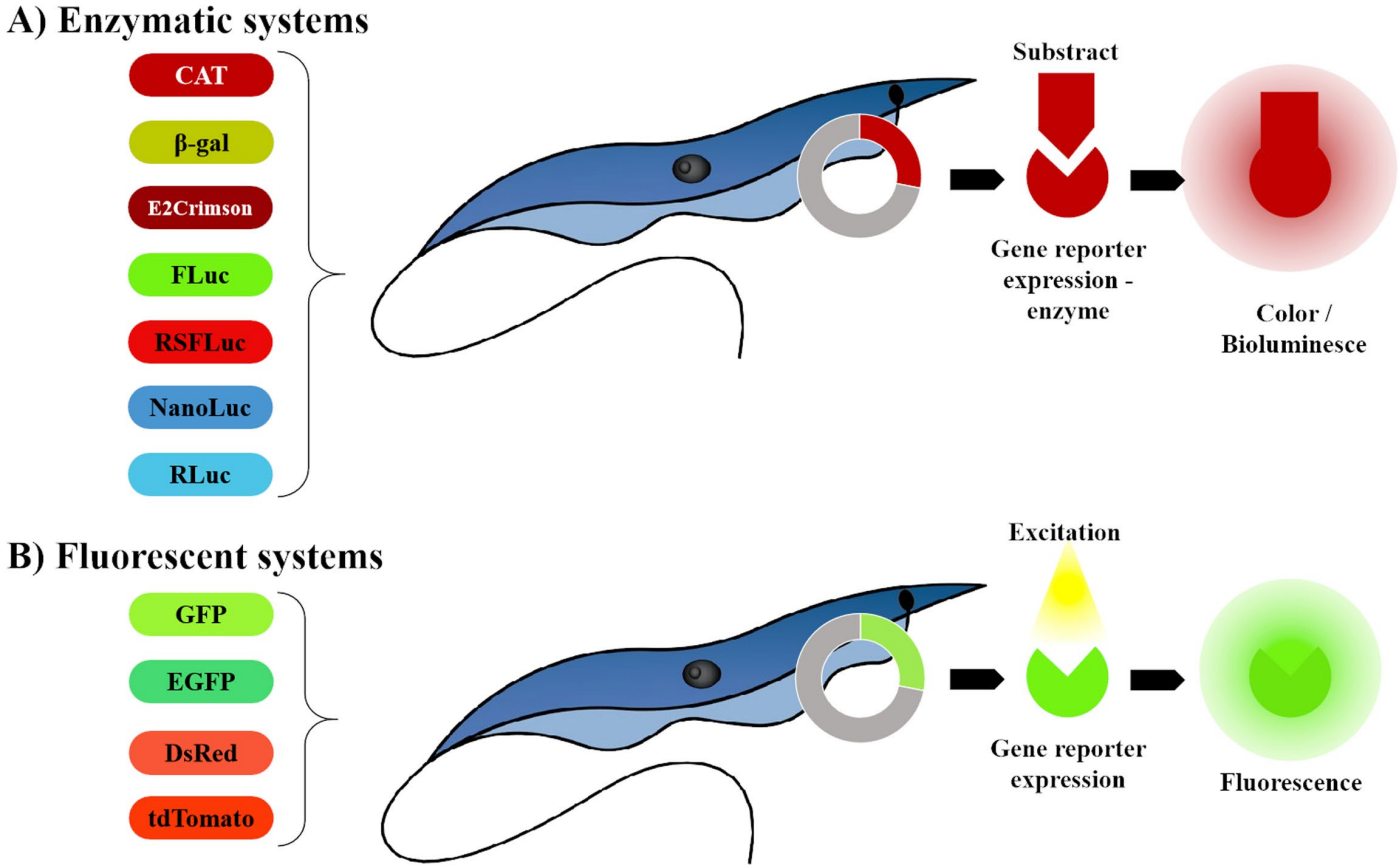
Representation of reporter systems categories, currently in use for anti- T. cruzi drug screening. **(A)** The enzymatic system depends on the enzyme reaction in the presence of the substract to express bioluminescence. **(B)** The fluorescent system requires no substrates, enzymes or cofactors to express fluorescence. The image was created by the authors using the following programs: AutoCAD 2024 - educational version, for drawing; WPS Office free version for assembling the figure (https://www.wps.com/) and GIMP 2.10.38 (gimp.org) for exporting with higher resolution (300dpi).

A reporter system codes for a detectable and quantifiable product in a living cell (20). For clarity, we have categorized reporter systems currently applied to *T. cruzi* into two groups: Enzymatic and Fluorescent (Figure 1): the enzymatic group, which includes chloramphenicol acetyltransferase (CAT), *β*-galactosidase (β-gal) reporters (21) and bioluminescent systems (20). The fluorescent group comprises green fluorescent protein (GFP); enhanced green fluorescent protein (EGFP); red fluorescent proteins such as Discosoma red fluorescent protein (DsRed) (20), tandem tomato (tdTomato) (22) and E2-crimson (23) (Table 1).

**Table 1:** Reporter genes systems highlighted characteristics, parasite strains and forms mentioned by the literature.

Reporter gene systems	Reporter gene	Parasite	Evolutive form	Highlighted characteristics	References
Enzymatic systems	CAT	*T. cruzi* CL.	Epimastigote.	Absent from mammalian cells; good reading of the signal in relation to the cellular background.	(71)
	β-gal	*T. cruzi* Tulahuen *β*-gal, clones C4 and CL B5 (DTU VI); *T. cruzi* CL-B5 (DTU TcVI).	Amastigote; trypomastigote.	Rapid quantification; adaptable to different devices and methods, including HTS; quantitative verification of the activity of specific promoters; visualization of cellular and subcellular activity in different tissues; identification of compounds against amastigotes.	(25, 29)
		*T. cruzi* CL-B5 (DTU TcVI).	Epimastigote.	Single step; consistent and reproducible.	(72)
		*T. cruzi* Dm28c/pLacZ (DTU TcI).	Amastigote; trypomastigote.	Standardization and validation of the colorimetric method; use in HTS; use of colorimetric readers.	(30)
		*T. cruzi*, Tulahuen strain (MHOM/CH/00/Tulahuen C2).	Amastigote; trypomastigote.	Use in murine model; parasites visible in tissues; quantification by microscopic scanning.	(31)
	FLuc	*T. cruzi* CL strain.		Transgenic parasites were used to evaluate the activity of anti-*T. cruzi*, in a short *in vivo* assay. The parasites provided results to long trials followed by immunosuppression.	(22)
		*T. cruzi* CL and Brazil, Dm28c-Luc clone.		The activity of compounds derived from naphthoquinones against *T. cruzi* was evaluated.	(73)
		*T. cruzi* Dm28c (DTU TcI) expressing FLuc.		Monitoring of *in vivo* infection in BALB/c mice. It allowed us to understand important aspects of the interaction between host and parasite.	(37)
	RS-FLuc	*T. cruzi* CL Brener.		Able to identify parasite persistence is the gastrointestinal tract.	(13)
		*T. cruzi* CL Brener.		Used with drug repositioning purpose.	(38)
		*T. cruzi* CL-Luc.		Higher sensitivity limit than that obtained by RT-PCR, over than a year after infection.	(39)
	NLuc	*T. cruzi* Colombiana (TcCOL-NLuc)		Use in an animal model and the use of the same strain in the study of infection of the human placenta.	(43)
	RLuc	*T. cruzi* CL-Luc	Trypomastigote.	Permit to check on the kinetics of infection in animal model and determine the precise sites of infection.	(11)
Fluorescent systems	GFP	Tulahuen (DTU TcVI) and Colombiana (Col.1.7G2, DTU TcVI)	Epimastigote.	Long duration of the signal. Suitable for use in *in vivo* models.	(51, 54)
		*T. cruzi* Tulahuen and JG (DTU TcII), Colombiana (DTU TcI), Col1.7G2 (derivada de Colombiana, DTU TcI), and CL Brener.	Amastigote; trypomastigote; epimastigote.	Constitutive expression.	(54)
		*T. cruzi* Dm28.	Epimastigote e amastigote.	Detection of proliferating parasites. Possibility of increasing the screening of compounds in HTS systems.	(50)
		*T. cruzi* K98-GFP.	Amastigote; trypomastigote; epimastigote.	Expresses fluorescence in all biological forms of the parasite. Method to determine the activity of compounds in just one step, by flow cytometry.	(55)
		*T. cruzi* expressing SMP1-1-GFP.	Amastigote.	Expresses fluorescence locally in the flagellum.	(74)
	EGFP	*T. cruzi* STIB980.	Epimastigote e amastigote.	Epimastigotes showed fluorescence 100 times higher than non-transfected ones, evaluated by flow cytometry.	(49)
		*T. cruzi* expressing EGFP and Ds-Red1-1.	Amastigote; trypomastigote; epimastigote.	The reporter genes did not affect the parasites. Epimastigotes have infectious characteristics. Possibility of using the proposed model for *in vitro* infection studies.	(49)
	mEGFP	*T. cruzi* Dm28c, Sylvio-X10 and Y strains.	Epimastigote; Trypomastigote.	Possibility of improving the expression of ectopic genes in *T. cruzi*. A versatile alternative to more sophisticated methods, such as CRISPR/Cas9.	(58)
	YFP	*T. cruzi* Y-GFP strain.	Amastigote; trypomastigote; epimastigote.	Use in drug repositioning strategies (carvedilol).	(20, 75)
	DsRed	*T. cruzi* Tulahuen, JG, Col1.7G2 derived from Colombiana and CL Brener.	Amastigote; trypomastigote; epimastigote.	First work describing fluorescent parasites, expressing GFP and RFP in animal tissue (BALB/c). It allows *in vivo* studies and the understanding of parasite invasion mechanisms, tissue tropism and genetic exchange mechanisms.	(54)
		*T. cruzi* CL transfected with DsRed and GFP.	Amastigote; trypomastigote; epimastigote.	Used in coinfection studies; allows 3D graphics with the exact location of the forms of *T. cruzi.*	(61)
		*T. cruzi* (GFP-G) and (DsRed-CL).	Amastigote; trypomastigote; epimastigote.	It allowed the first images of individualized fluorescent amastigotes in nests, in the tissues of various murine organs, in addition to intermediate forms and ex vivo motile trypomastigotes, obtained by confocal microscopy.	(62)
	tdTomato	*T. cruzi* CL tdTomato.		Screening of potential anti-*T. cruzi*, no need for cell fixation or permeabilization, scalable to 384-well format, allows the development of HTS.	(64)
		*T. cruzi* CL tdTomato.		Screening of potential compounds. Development of *in vitro* and *in vivo* tests applicable to HTS. Detectable by microscopy and flow cytometry. Possibility of quantifying fluorescence by plate reader. It allowed monitoring replication at the site of infection and quickly determining the effectiveness of treatment.	(22)
	E2-Crimson	*T. cruzi* expressing E2-Crimson (*TcTREX*-Crim).	Amastigote; trypomastigote; epimastigote.	Applications for imaging deep tissues *in vivo*.	(23)
		*T. cruzi Silvio* X10/7 A1.	Epimastigote; Trypomastigote.	Permit live-imaging assay of intracellular forms of *T. cruzi* to determine the rate-of-kill (RoK) profile of evaluated compounds.	(66)

## 2 Enzymatic systems

### 2.1 Chloramphenicol acetyltransferase

Chloramphenicol acetyltransferase (CAT), the first reporter used to assess mammalian transcriptional activity of mammalian is stable and absent in mammalian cells (24). Early assays were lengthy, costly, and required radioactive tracers (20), later replaced by non-radioactive fluorescent or immunosorbent methods (24). Thus, this system proves to be inadequate for automated analyses and high-throughput screening, which are essential for the discovery of new drugs for Chagas disease.

### 2.2 *β*-galactosidase

The *Escherichia coli* lacZ gene encodes β-galactosidase (β-gal), the first reporter gene used in *T. cruzi* for *in vitro* screening (21). Buckner et al. (25) developed the Tulahuen β-gal strain (clone C4 and CL clone B5, DTU VI), detectable in host cells, enabling compound screening against intracellular parasite by colorimetric assay (25), thus allowing the measurement of parasite proliferation under compound exposure.

The Tulahuen strain is compatible with microplate readers assays (12) and adaptable to High-Content (26) and HTS systems (27), as demonstrated by GlaxoSmithKline HTS campaign, which identified 500 non-cytotoxic compounds (28). Broad Institute MLPCN *T. cruzi* Inhibition Project (29) used Tulahuen *β*-gal strain with the GalScreen luminescent reporter system on 303,224 compounds, identifying 4,394 hits (12).

Colorimetric reporters may interfere with enzymatic detection, but fluorometric or luminescent readings can resolve it (30). Gulin et al. (30) minimized interference by removing the supernatant before adding Chlorophenol red-*β*-D-galactopyranoside (CPRG) substrate and validated an *in vitro* assay using the transfected *T. cruzi* Dm28c/pLacZ strain (DTU TcI). The findings enphasized both the reliability and reproducibility of the assay and its suitability for HTS systems.

Also, β-galactosidase enables the study of parasitism throughout all CD stages in animal models: using 5-bromo-4-chloro-3-indolyl-β-D-galactopyranoside (X-gal) as a substrate, parasites become visible after fixation and blue staining, allowing quantification by microscopic scanning post-euthanasia (31). Although well-stablished and reliable, the lacZ system is limited by the requirement for cell lysis in order to assess enzymatic activity, preventing real-time analysis.

### 2.3 Luciferase systems

Luciferases catalyze light production by converting a supplemented substrate (16). Their efficient oxidation, bioluminescence and quantifiable activity make them valuable as reporters (32), particularly in *T. cruzi* transfection, where they reduce interference from colored compounds in HTS assays (12). *In vivo*, luciferase is ideal since animals lack endogenous activity, enabling reliable, multiple measurements. However, for identifying or quantifying reporter-expressing cells, fluorescent proteins are preferable remain more suitable (33).

The studies here presented enabled more accurate host–parasite interaction analysis in deep tissues and drug screening, and demonstrated the reporters effectiveness in assessing parasite replication. However, those systems require specific substrate addition, with possible limited bioavailability. It also requires specialized bioluminescent detection equipment.

#### 2.3.1 Firefly luciferase

Firefly luciferase (FLuc) emerges as valuable reporter for anti-*T. cruzi* drug development (18) as it detects trace ATP levels and emits light efficiently through mammalian tissues (34). Although FLuc requires luciferin addition (12), it allows *in vivo* imaging without cell lysis, supporting whole-animal and HTS assays (21). However, its sensitivity decreases in deep tissues due to its <600 nm emission, which is absorbed by hemoglobin—a limitation that can be addressed with reporter emitting above 600 nm (35). Also, FLuc can be inhibited by small molecules structurally related to D-luciferin, such as benzothiazoles, benzimidazoles, benoxazoles, and biaryl oxadiazoles, resulting in false positives during inhibition assays (36).

Despite these limitations, significant advance has been achieved. Canavaci et al. (22) used a luciferase-expressing CL strain *T. cruzi* to infect Balb/c mice and developed a 12-day assay comparable to traditional 80-days protocols with immunosuppression (22), representing an advancement that allows for rapid and non-invasive assessment of the drug’s efficacy through bioluminescence.

In another work, Henriques et al. (37) tracked the progression of *in vivo* infection using *T. cruzi* (Dm28c-luc) in BALB/c mice, identifying new infection sites for the first time: the luminescent signal was observed at the inoculation site, reaching the peritoneal cavity 1 day post-infection and spreading to abdominal organs and adjacencies, shedding light on both the pathology and the interaction between parasite and host. Studies like these advanced the understanding of *T. cruzi*’s pathogenesis with importance for new drugs development.

#### 2.3.2 Red-shifted firefly luciferase

Red-shifted firefly luciferase (RS-FLuc) is a variant of luciferase with enhanced sensitivity and stability, offering improved visualization in deep mammalian tissues (38); it has been used to identify *T. cruzi* tropisms in mice, as reported by Lewis et al. (13), who integrated the thermostable red-shifted luciferase gene into the parental CL Brener strain, allowing the monitoring of animals over a year, with a detection limit of 100 parasites, and revealed gastrointestinal tissue as primary site of parasite persistence.

The luciferase permitted to assess posaconazole’s efficacy against acute and chronic CD, Francisco et al. (38) inoculated mice with CL-Brener strain and evaluated them using an *in vivo/ex vivo* imaging system after an strategic use of cyclophosphamide-induced immunosuppression to uncover residual *in vivo* infection, in which posaconazole demonstrated inferior performance compared to benznidazole in both infection phases. In other study, Calvet et al. CL Brenner strain expressing red-shifted luciferase (*T. cruzi* CL-luc) which enabled the detection of live parasites in mouse tissues surpassing RT-PCR sensitivity, for over a year post-infection (39), also enabling drug efficacy tracking through different phases of the CD.

#### 2.3.3 NanoLuc

NanoLuc (NLuc) derived from the luciferase of *Oplophorus gracilirostris* (A. Milne-Edwards, 1881) (40) (Decapoda, Oplophoridae) and furimazine (41), is used in *in vivo* Bioluminescence Imaging due to its sensitivity and intense luminescence (42), enabling multiplexing with longer-wavelength reporters (41). Colombian strain of *T. cruzi* expressing NLuc (TcCOL-NLuc) has been applied to study placental crossing in 3D cell culture models (43), providing insights into tissue tropism and highlighting NLuc’s utility for ADMET evaluation in drug screening, under physiologically relevant conditions.

#### 2.3.4 Renilla luciferase

*Renilla reniformis* (Pallas, 1766) (44) (Scleralcyonacea, Renillidae) (RLuc) luciferase catalyzes the oxidation of coelenterazine producing “bioluminescence, coelenteramide and CO_2_” (32), and serves as a reporter for bioluminescent imaging in animal models; however, its sensitivity depends on the depth of the tissue investigated (45). The applications of RLuc are discussed in section 4 (Multiple Gene Reporter Systems), to which we direct the reader for further details.

## 3 Fluorescent systems

Green fluorescent protein (GFP) chromophore emits light without needing cofactors or substrates (46), however all fluorescent proteins photobleach under prolonged excitation, making photostability essential repetitive imaging experiments (47). Mutations in the GFP gene produce reporters with varied colors and intensities based on aminoacid sequences (48). This led to the development of *T. cruzi* strains enhanced green fluorescent Protein (EGFP) (49) and red fluorescent proteins, such as *Discosoma striata* (Corallimorpharia, Discosomidae) (DsRed) (21), tandem tomato fluorescent protein (tdTomato) (22) and E2-crimson (23) which have been applied in drug screening for CD (50). Fluorescent proteins enable real-time imaging without cell lysis but require specialized equipment and may be affected by tissue autofluorescence.

### 3.1 Green fluorescent protein

Encoded by a single gene, requiring no substrates, enzymes or cofactors (34), GFP is a low cost, non-toxic reporter for *in vitro* and *in vivo* imaging (23), detectable via microplate readers, fluorescence microscope, Fluorescence-Activated Cell Sorter (FACS) or fluorimetry (21). Da Rocha et al. (51) stably expressed GFP in Tulahuen (DTU TcVI) and Col.1.7G2 (DTU TcVI) epimastigotes, with persistent signal for over 5 weeks without drug selection. Despite its stability, GFP’s excitation can damage cells (52) and detection is limited to ~1 mm depth from the surface, suitable for small/transparent models (53).

Nevertheless, GFP-expressing strains like Tulahuen and JG (DTU TcII), Colombiana (DTU TcI), Col1.7G2 (DTU TcI) and CL Brener hybrids have shown infectivity *in vitro* and *in vivo*, as seen in confocal and fluorescence microscopy (54).

The pBEX/GFP strain, derived from Dm28 (DTU TcI) by Kessler et al. (50), exclusively expresses GFP in replicative forms of *T. cruzi*, allowing their growth tracking. Kessler et al. (50) also validated a screening method based on GFP signal intensity for HTS. Miranda et al. (55) developed the K98-GFP strain (DTU TcI) fluorescent in all *T. cruzi* stages facilitating single-step screening. GFP mutations have expanded emission spectra, improving signal quality and broadening application possibilities (48).

### 3.2 Enhanced green fluorescent protein (EGFP and mEGFP)

EGFP displays stronger fluorescence than GFP (48). Florêncio-Martínez et al. (56) used *T. cruzi* expressing EGFP and Ds-Red1-1 to investigate the infection process showing that reporter expression did not affect parasites infectivity. All forms infected NIH-3 T3 fibroblasts with similar kinetics, validating the model for *in vitro* infection studies (56).

Fesser et al. (49) monitored EGFP-expressing *T. cruzi* STIB980 amastigotes every 4 h for 6 days in mouse embrionic fibroblasts, using high-content imaging and. Pharmacodynamic analysis and flow cytometry revealed EGFP-expressing epimastigotes exhibited 100-fold higher autofluorescence than non-transfected cells (49). This temporal assessment of parasite growth is key in evaluating compounds effect on parasite replication, particularly as amastigotes represent the main stage for therapeutic targeting (9, 14) and epimastigotes once considered non-infective are now recognized for their potential in infection studies (57).

Niemirowicz et al. (58) further advanced the genetic engineering of *T. cruzi* by comparing conventional multi-mRNAs vectors to those based on 2A self-cleaving peptides for mEGFP expression in Dm28c, Sylvio-X10 and Y strains, offering a more efficient alternative to CRISPR/Cas9 for endogenous gene labeling and expanding the toolkit for reporter-based drug screening platform.

### 3.3 *Discosoma* sp. (DsRED)

Red fluorescent proteins (RFPs) expanded the imaging spectrum beyond GFP, with the first RFP exhibiting excitation/emission peaks at 555/585 nm (59). DsRed, isolated from (*Discosoma* sp.) (20), was the first fluorescent protein from a non-photosynthetic organism and is widely used due to its high photostability and compatibility with confocal microscopy and flow cytometry (52). Pires et al. (54) engineered *T. cruzi* strains expressing RFP or GFP (pROCKRFPNeo and pROCKGFPNeo vectors) in epimastigotes of Tulahuen, Col1.7G2 and CL Brener. Fluorescence remained stable for over 6 months and dual-color imaging revealed coinfection of host cells by different strains, enabling studies on invasion dynamics, tissue tropism and genetic exchange.

Usign *T. cruzi* CL strain coexpressing DsRed and GFP, researchers visualized parasite differentiation within *Leishmania amazonensis* Lainson & Shaw, 1972 (*L. amazonensis*) (60) revealing that metacyclic trypomastigotes developed into amastigote-like forms, failing to reach the cytosol (61). These findings highlight species-specific requirements for intracellular differentiation, offering insights into host–parasite relations that may critically interfere in drug targeting.

Ferreira et al. (62) observed *T. cruzi in vivo* in BALB/c or C57BL/6 mice infected with G strain (DTU TcI) parasites transfected with GFP (GFP-G) or CL strain (DTU TcVI) trypomastigotes transfected with DsRed (DsRed-CL). The method provided the first images of fluorescent amastigotes in tissue nests as well as intermediate forms and motile *ex vivo* trypomastigotes, visualized by confocal microscopy (62).

These studies demonstrate the potential for direct monitoring of tissue infection using DsRed reporter, a critical step in evaluating the efficacy of new drug candidates, and potential understanding of the infection, refining the design of preclinical assays.

### 3.4 Tandem dimeric tomato red fluorescent protein

Among the brightest and photostable fluorescent proteins, tdTomato (a DsRed variant) exhibits excitation/emission peaks at 554/581 nm (63). Bustamante et al. (64) used the *T. cruzi* CL tdTomato strain for anti-*T. cruzi* screening, highlighting its compatibility with HTS abd the advantage of not requiring cell fixation or permeabilization (64).

Canavaci et al. (22) developed a *T. cruzi* CL tdTomato strain constitutively expressing tdTomato suitable for *in vitro* and *in vivo* HTS. The strong fluorescent signal across all stages enabled replication monitoring and rapid treatment efficacy assessment by microscopy, flow cytometry and plate reader (22). *Trypanosoma cruzi* tdTomato strains allow non-invasive *in vivo* monitoring of parasitemia tracking and parasite distribution via fluorescent imaging (65).

### 3.5 E2-Crimson

E2-Crimson, a DsRed fluorescent protein is non-toxic photostable rapidly maturing, and suitable for live-animal imaging, flow cytometry and stimulated emission depletion (STED) microscopy (23). Aiming applications for imaging deep tissues *in vivo* infected by *T. cruzi*, Goyard et al. (23) developed *T. cruzi* Y expressing E2-Crimson fluorescence (TcTREX-Crimson), offering another tool for drug screening, particularly in the *in vivo* context.

In other hand, by combining an *in vivo* imaging assay that allows for real-time, detailed evaluation of parasite clearance without invasive techniques and the use of cardiomyocytes with nuclear EGFP, Svensen et al. (66) enabled visualization of the parasite (Tc-X10/7-E2Crimson) and host interaction as well as the effect of the compound on target cells, leading to a more precisa, efficient drug screening.

## 4 Multiple gene reporter systems

Reporter systems with different substrates, spectra and emission kinetics can be used simultaneously in the same animal without cross-reaction, enabling distinct emissions and timeline readings (34), a allowing monitoring of parasite behavior and drug efficacy without interference between one another.

EGFP and DsRed1-1 fluorescent proteins were used as *T. cruzi* reporters in Florêncio-Martinez et al. (56) to study infectious process in live cells. This model provided insights into molecular mechanisms of intracellular microorganism infection (56), permitting real-time observation of the infection progress through treatment, *in vitro* and *in vivo*, as shown by Canavaci et al. (22).

Costa et al. (11) investigated *T. cruzi* role in CD progression by generating a reporter strain, CL-Luc, incorporating a red-shifted luciferase/GFP fusion protein (Luc-mNeonGreen). Luminescence and fluorescence enabled monitoring of infection kinetics, infection sites, and parasite–host interactions at cellular level (11). The strain was further modified with CRISPR/Cas9 to generate null parasites with fluorescence (11) enabling analysis of gene function and drug efficacy at molecular levels.

Taylor et al. (67) used *T. cruzi* CL-Luc:Neon strains expressing chimeric bioluminescent and fluorescent protein to visualize individual parasites in mouse tissue and investigate replication in host cells. Ward et al. (68) developed a transgenic *T. cruzi* expressing bioluminescent and fluorescent fusion proteins, proposing a model for heart disease development during chronic phase. Precise information on parasite persistence site was needed. Using murine tissue, ex vivo imaging and confocal microscopy, they visualized host cells infected with two strains: *T. cruzi* CL-Luc:Neon, a CL Brener clone (DTU TcIV) expressing red-shifted luciferase linked to mNeonGreen; and the JR Clone (DTU TcI) expressing red-shifted luciferase (68).

Dual systems have gained space in drug repositioning: Rivero et al. (69) generated the Tulahuen Luc-mNeonGreen strain (DTU TcVI), expressing a double reporter gene, and infected mice to characterize carvedilol’s efficacy as a promising hit.

Olmo et al. (70) developed a panel of transfected *T. cruzi* strains, expressing bioluminescent/fluorescent fusion proteins—a tool expected to enhance data from experimental infection models, *in vitro* and *in vivo*, and enable studies of mixed infections in CD drug development (70).

## 5 Conclusion

In conclusion, based on the reviewed literature, genetically modified parasites expressing fluorescent and luminescent proteins represent a promising approach for real-time monitoring of infection and treatment response. The simultaneous use of multiple reporter systems, despite potential challenges related to cost and optimization, appears to be a promising strategy to combine the advantages of different techniques while reducing their limitations. These reporter systems facilitate compounds screening with trypanocidal potential and enhance the analysis of parasite–host interactions, significantly contributing to the development of new therapies against *T. cruzi*.

Transgenic parasites, as crucial tool for drug discovery, offers benefits such as reduced costs, time and labor. This review correlates the evolutionary forms of transgenic *T. cruzi*, reporter genes, and their applications as valuable tools for screening drug candidates against CD, providing a framework for future research models.

Advancements in the application of transgenic parasites mark a significant step forward in pharmacological research. However, continuous methodological optimization is necessary to maximize their potential in the development of new treatments for CD.
